# An Uncommon Complication of Transoral Incisionless Fundoplication

**DOI:** 10.7759/cureus.102661

**Published:** 2026-01-30

**Authors:** Abu F Abbasi, Sultan Ahmed, Syed Y Ali, Adam Geibel, Luqman Baloch, Ahmet Sakiri, Altaf Dawood, Naser Khan, Thayer Hamoudah

**Affiliations:** 1 Gastroenterology, Mercyhealth Graduate Medical Education (GME) Consortium, Rockford, USA; 2 Internal Medicine, Mercyhealth Graduate Medical Education (GME) Consortium, Rockford, USA; 3 College of Medicine, Allegheny Health Network, Erie, USA; 4 College of Medicine, Lake Erie College of Osteopathic Medicine, Erie, USA; 5 Internal Medicine, Lake Erie College of Osteopathic Medicine, Erie, USA; 6 Internal Medicine, University of Illinois Chicago, Rockford, USA

**Keywords:** gastroesophageal reflux disease (gerd), hemorrhage, iatrogenic complication, transoral incisionless fundoplication (tif), upper endoscopy

## Abstract

In this report, we present a case in which a patient developed hemodynamic instability and melena two days after transoral incisionless fundoplication (TIF), a procedure perceived to have a safer adverse event profile. After the patient was stabilized, repeat esophagogastroduodenoscopy showed a Forrest IIa ulcer on the gastric cardia at the suture site, which was cauterized and resolved. This case highlights that significant hemorrhagic events can occur after a TIF procedure and reviews approaches to anticipate this rare occurrence and steps to prevent it.

## Introduction

Gastroesophageal reflux disease (GERD), the transmission of acidic and particulate contents from the stomach into the esophagus, can occur due to structural, mechanical, and/or physiologic causes. With a 77.5% increase in prevalence from 1990 to 2019, chronic reflux has been associated with dysplastic changes in the esophageal mucosa, with the potential to progress to a premalignant condition known as Barrett’s esophagus [1]. Management consists of lifestyle and dietary changes. Refractory symptoms are addressed with pharmacologic therapy such as H2 receptor antagonists (H2RAs) and proton pump inhibitors (PPIs). Per the American Gastroenterological Association, persistent symptoms qualify patients for further assessment and procedural candidacy evaluation, including endoscopic evaluation for pathologic GERD, achalasia, and/or hypomotility [2]. For patients with evidence of refractory reflux in the setting of lower esophageal sphincter (LES) laxity, several options exist for endoscopic and surgical intervention, including Nissen fundoplication or transoral incisionless fundoplication (TIF). TIF has gained popularity due to a perceived, comparatively advantageous adverse-event profile compared to Nissen fundoplication. However, rare complications such as life-threatening hemorrhage can still occur and may deter patients. We present the case of a 39-year-old male who developed major hemorrhagic complications two days after TIF. This case highlights a potentially fatal complication of TIF and weighs it against other options for patients seeking relief from long-standing GERD.

## Case presentation

**Figure 1:**
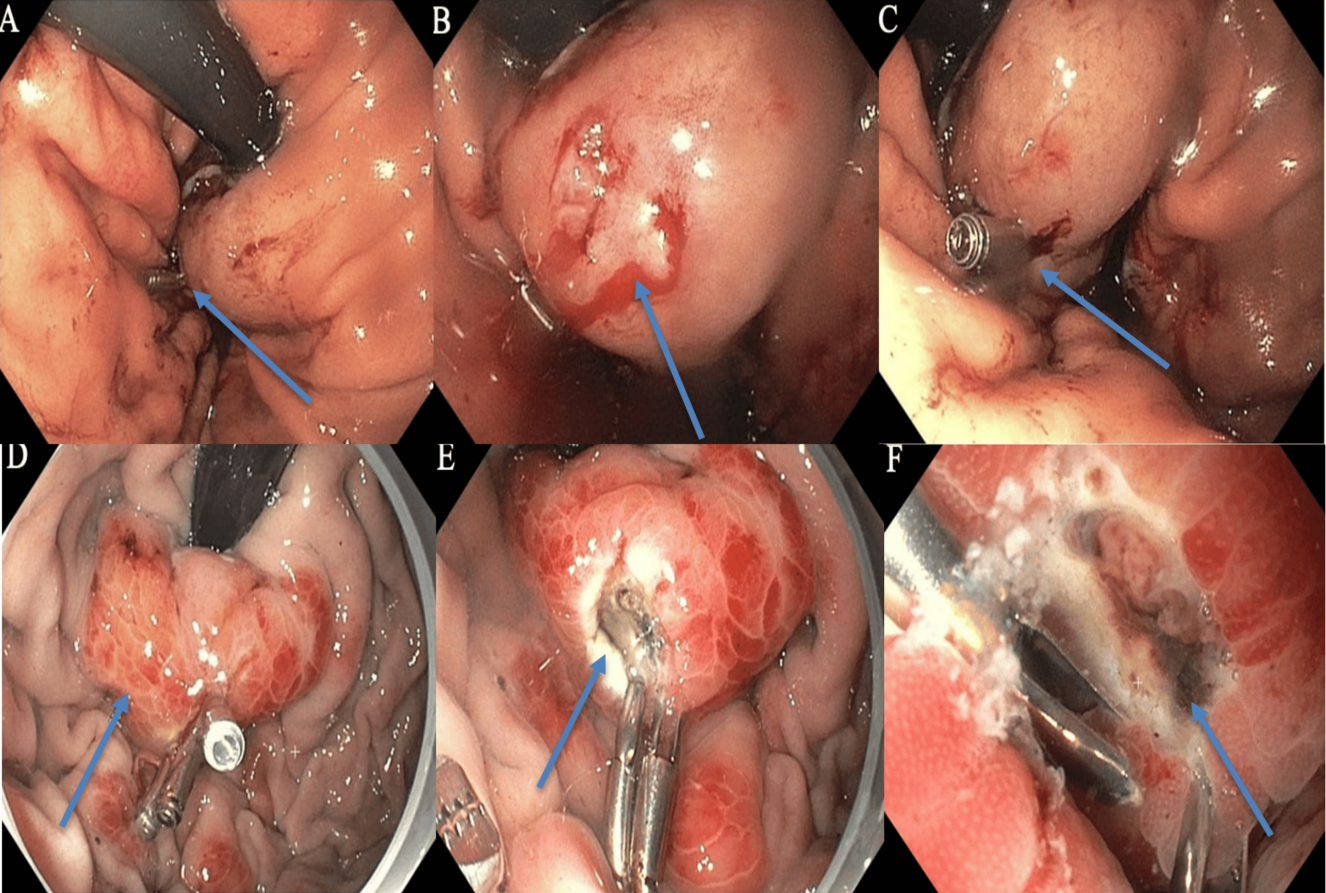
Endoscopic findings before and after admission. Images (A-C) show the initial endoscopic findings during TIF, prior to admission, with clip placement. Forrest class IIa ulcers in the gastric cardia are shown, and the arrows in (A) and (C) indicate the clips in place. Image (B) shows a site of mucosal bleeding prior to clip placement. Images (D-F) show the repeat EGD, with visible non-bleeding vessels at the site of the previously placed hemostatic clips. The arrow in (D) indicates erythematous tissue, which was the likely source of bleeding. Images (E) and (F) show the site after cautery. TIF: Transoral Incisionless Fundoplication; EGD: Esophagogastroduodenoscopy.

A 39-year-old male with a past medical history of GERD presented to the ER with dizziness, presyncope, and melena two days after undergoing a TIF procedure. His medication history included only pantoprazole. In the ED, he was diaphoretic, with a heart rate of 110 bpm and a blood pressure of 60/37 mmHg; the remaining vital signs were stable. He was given 30 cc/kg of IV fluids, piperacillin-tazobactam, vancomycin, and pantoprazole for undifferentiated septic vs hemorrhagic shock on arrival. Labs were significant for Hgb of 12.6 g/dL (baseline 14 g/dL), blood urea nitrogen (BUN) of 34 mg/dL, and lactic acid of 2.5 mmol/L. Vitals stabilized after resuscitation. Rectal exam was positive for melanotic stool. The patient was given two units of packed RBCs, continuous IV fluids, and a pantoprazole drip. Upon review of the operative note, endoscopy two days prior was notable for completion of a Hill Grade/AFS Grade II flap valve status post successful TIF with 24 total fasteners, with significant findings of two areas of focal oozing in the cardia, which were treated with soft coagulation using a coagulation grasper and two hemostatic clips. He was noted to be on pantoprazole pre- and peri-procedurally. After stabilization, the patient underwent repeat esophagogastroduodenoscopy, which revealed a single Forrest IIa ulcer at the gastric cardia with a visible non-bleeding vessel. This was cauterized via bipolar cautery. Additional hemostatic clips were placed. Two previously placed clips were intact (Figure 1). The patient was discharged in stable condition on pantoprazole therapy.

## Discussion

The reflux of gastric contents into the distal esophagus, as seen in GERD, can induce dysplastic changes if prolonged. The most implicated cause of reflux is laxity of the LES; reasons for this include intrinsic LES relaxation or a secondary cause such as a sliding hiatal hernia [3,4]. Although GERD is primarily a clinical diagnosis, endoscopy can be considered in select populations; biopsies showing esophagitis can confirm the diagnosis [5]. Initial management involves diet and lifestyle modifications, with step-up to pharmacotherapy. Antacids, H2RAs, and PPIs are the most common pharmacologic treatments, while other options, such as prokinetics, baclofen, and sucralfate, have shown potential benefits for GERD but are not consistently effective. When lifestyle changes and pharmacotherapy are insufficient, preoperative testing should be performed, and providers can consider procedural intervention [3,6].

With a prevalence ranging from 18% to 27% in the United States, GERD affects a significant portion of the population [7]. Pursuit of surgical intervention can be an appropriate avenue for patients with refractory GERD, esophagitis, Barrett’s esophagus, reflux with resultant stricture formation, or concern for prolonged PPI use. Although the approximate population of patients with refractory GERD is not clear, less than 1% of the documented population elect for surgical intervention annually [8]. Candidacy is determined by first undergoing an EGD, barium esophagram, or esophageal manometry to assess the anatomy of the gastroesophageal (GE) junction [7]. After initial evaluation, options for intervention include Nissen fundoplication, magnetic sphincter augmentation, Roux-en-Y gastric bypass, and endoscopic solutions such as radiofrequency anti-reflux treatment [9]. Laparoscopic fundoplication, or Nissen fundoplication, has been considered the gold standard surgical intervention, but other approaches have gained popularity in recent years. TIF is an endoscopic approach with a similar procedural profile, and it is increasingly preferred by providers. Primary differences include the approach and the degree of circumferential tightening of the gastric anatomy (various options for Nissen vs 270 degrees for TIF). TIF carries a lower risk of complications such as bleeding and infection, while Nissen is associated with fewer wound and respiratory complications. However, both approaches demonstrate similar efficacy [10,11].

Fewer than 30,000 Nissen procedures are performed annually in the U.S. Patients with prior upper abdominal surgery, a BMI >35, or esophageal dysmotility are generally not considered surgical candidates [7,12]. Despite demonstrated efficacy, concerns remain regarding long-term failure and side effects such as bloating and flatulence [13-16]. Serious adverse events include <1% mortality, <2.5% conversion to open surgery, and a 5-20% rate of immediate postoperative morbidity. Common causes of mortality include gastrointestinal hemorrhage due to disruption of short gastric vessels, gastric necrosis, perforation, cardiac arrest, respiratory complications, and pulmonary embolism [15,16]. In comparison, approximately 30,000 TIF procedures have been performed worldwide since its introduction in 2006, and its popularity has grown [13,17]. In a meta-analysis of 781 TIF patients, the incidence of adverse events was 2.4%, including perforations, bleeding, pneumothorax, antibiotic use, and severe epigastric pain, with only one reported death [18]. Another study involving 550 patients reported a 3.2% rate of serious adverse events, with bleeding being the most common (1.1%) [12,19]. Due to its less invasive nature, TIF is associated with fewer complications and shorter hospital stays [9,20]. While both procedures aim to correct LES laxity and reduce reflux, the decision between Nissen and TIF should be guided by patient-specific factors. TIF is generally preferred in patients who have not responded to medical therapy and have a small or absent hiatal hernia; it is contraindicated in those with Barrett’s esophagus or a large hiatal hernia [21]. Nissen fundoplication, on the other hand, is indicated in cases of severe refractory GERD, large hiatal hernias, normal motility, and GERD complications such as Barrett’s esophagus or peptic stricture. Partial approaches may benefit patients with esophageal hypomotility or impaired peristalsis by reducing the risk of postoperative dysphagia [22]. Although both procedures carry a risk of bleeding, the lower number of reported TIF cases, along with the relatively low percentage of adverse bleeding events, suggests a favorable post-procedural complication profile. Although more cases are needed to strengthen the data compared with Nissen fundoplication, this may be beneficial for patient populations that refuse blood transfusions. In a case involving treatment of GERD in a Jehovah’s Witness, TIF was chosen over Nissen fundoplication due to the perceived diminished risk of adverse bleeding events requiring blood transfusion, with successful outcomes [17].

In this patient’s case, given his elevated BMI (34.44 kg/m²), preference for avoiding a laparoscopic approach, and joint decision-making with his gastroenterologist, TIF was chosen for his management. His source of bleeding was at the previously clipped ulcer sites, which suggests either inadequate hemostasis initially or iatrogenic bleeding due to the procedure. Although he was appropriately resuscitated and the source of bleeding was addressed, this case suggests several points for consideration. When performing TIF, if ulcers are noted, endoscopists might consider delaying the procedure by a few weeks to allow ulcer healing after hemostasis. His gastric ulcer was noted to be Forrest IIa, which, per the classification system, has a 43% chance of re-bleeding [7,23]. Therefore, ulcer classification may help stratify this decision-making, along with the presence of active bleeding on initial surveillance. Another consideration is avoiding sites of noted ulcers as fastening points when wrapping the gastric fundus around the LES. In a study evaluating the 6-month safety and efficacy of TIF, 8 of 69 patients experienced significant adverse effects. Six of the eight patients received only two staples during the procedure. Changes were made to add additional stapling to reduce events. Another change was prophylactic therapy to prevent postoperative retching, as retching could stress and weaken the staples. Confirmatory X-rays were also obtained to ensure no leakage [14,24]. Other studies have used additional endoscopic clips in tandem with fibrin glue to reduce the risk of peri- or post-procedural bleeding [7,23]. Regarding technique, specific observations have been made about procedural approaches and their impact on outcomes. The helical fastener has been suggested as a common source of bleeding, with recommendations such as deploying endoclips in a retroflex position to manage bleeding at the edge of the plication. Other standardized recommendations include firing one fastener at a time to reduce the number of clips needed, properly positioning the device to prevent misfiring (and thereby reducing repeat attempts), and minimizing the risk of laceration [15,24]. Procedure success may also be affected by provider experience; in a study in which no adverse events were recorded, all providers had successfully completed 20 procedures beforehand [6,16]. As previously mentioned, an increase in cases, given the growing popularity of this procedure, would enable a better understanding of its utility.

## Conclusions

TIF is a minimally invasive procedure aimed at managing refractory GERD in patients whose symptoms cannot be adequately controlled with medication or lifestyle modifications. Compared with Nissen fundoplication, TIF has demonstrated similar outcomes and efficacy, with differences in the type and incidence of complications. Although these risks are exceedingly low, complications must be recognized and managed effectively to achieve good outcomes. Several studies have suggested technical modifications to prevent adverse events. This case aims to bolster the available evidence and to encourage provider vigilance when weighing potential benefits and harms. Although further data are needed to support one intervention over another, physicians should engage in patient education and shared decision-making to determine the most appropriate approach for each patient.
